# Personals

**Published:** 1918-09

**Authors:** 


### PERSONAL

Announcement of removal, travel, and other matters of interest are requested. Please report errors in the listing of any physician in the State and other directories, that we may co-operate with the State Society in securing a correct list.

---

Dr. Franklin W. Barrows of the Buffalo Health Dept. has been appointed Assitant State Medical Inspector of Schools, in the State Dept. of Education and will reside in Albany.

Dr. M. M. Cole, attending physician to the Niagara County

Infirmary at Lockport, has resigned to accept the position of Assistant Physician at the Soldiers' Home, Bath.

Dr. Franklin C. Gram, Acting Health Commissioner of Buffalo, celebrated a quarter of a century of service with the Health Dept., Aug. 7.

Dr. Frederick Hayes motored through .the Adirondacks in August.

Dr. Floyd S. Crego has returned from Keuka Lake.

Dr. George Gorrill has been appointed superintendent of the Buffalo State Hospital, succeeding Dr. Arthur W. Hurd, who resigned on March 18th. Dr. Gorrill's appointment is announced by the state civil service commission and he is now in charge of the Forest Avenue hospital for the insane. The new superintendent has been a resident physician of the institution for seventeen years. For the last seven years he has been first assistant to the superintendent and since the resignation of Dr. Hurd has been in charge of the hospital. Dr. Hurd left the hospital soon after presenting his resignation.

Dr. Edward H. Mehl announces the removal of his office to 127 Allen St. Practice confined to diseases of the ear, nose and throat.

